# The exocyst at the interface between cytoskeleton and membranes in eukaryotic cells

**DOI:** 10.3389/fpls.2013.00543

**Published:** 2014-01-02

**Authors:** Lukáš Synek, Juraj Sekereš, Viktor Žárský

**Affiliations:** ^1^Laboratory of Cell Biology, Institute of Experimental Botany, Academy of Sciences of the Czech RepublicPrague, Czech Republic; ^2^Laboratory of Plant Cell Biology, Department of Experimental Plant Biology, Faculty of Science, Charles University in PraguePrague, Czech Republic

**Keywords:** exocyst, actin cytoskeleton, microtubule cytoskeleton, phospholipids, myosin, small GTPases, Exo70, secretion

## Abstract

Delivery and final fusion of the secretory vesicles with the relevant target membrane are hierarchically organized and reciprocally interconnected multi-step processes involving not only specific protein–protein interactions, but also specific protein–phospholipid interactions. The exocyst was discovered as a tethering complex mediating initial encounter of arriving exocytic vesicles with the plasma membrane. The exocyst complex is regulated by Rab and Rho small GTPases, resulting in docking of exocytic vesicles to the plasma membrane (PM) and finally their fusion mediated by specific SNARE complexes. In model Opisthokont cells, the exocyst was shown to directly interact with both microtubule and microfilament cytoskeleton and related motor proteins as well as with the PM via phosphatidylinositol 4, 5-bisphosphate specific binding, which directly affects cortical cytoskeleton and PM dynamics. Here we summarize the current knowledge on exocyst-cytoskeleton-PM interactions in order to open a perspective for future research in this area in plant cells.

## THE EXOCYST AS A REGULATORY HUB IN THE ACTIVE CELL CORTEX

**FIGURE 1:**
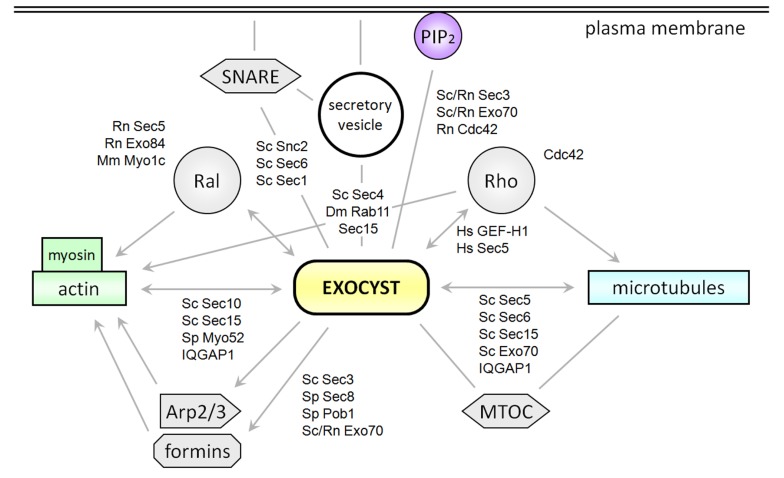
**Interactions of the exocyst complex with the cytoskeleton, plasma membrane, and associated proteins.** Scheme of exocyst interactions described in the text, including the key players Ral and Rho GTPases, SNARE proteins, formins, and the Arp2/3 actin-nucleating complex, microtule-organizing center (MTOC) and phosphatidylinositol 4, 5-bisphosphate (PIP_2_) at the plasma membrane. Dm, *Drosophila melanogaster*; Mm, *Mus musculus*; Hs, *Homo sapiens* Rn, *Ratus norwegicus*; Sc, *Saccharomyces cerevisiae*; Sp, *Schizosaccharomyces pombe*.

Polarized surface growth in eukaryotic cells involves interactions between the cytoskeleton and membrane transport pathways. The last steps of the secretory pathway taking place in the vicinity of the plasma membrane (PM) are regulated by an array of small GTPases, the exocyst tethering complex, and SNARE proteins. The exocyst is a protein complex comprising eight subunits (Sec3, Sec5, Sec6, Sec8, Sec10, Sec15, Exo70, and Exo84) engaged in docking and tethering of secretory vesicles, providing a spatial and temporal regulation of exocytosis (Hsu et al., 1996; TerBush et al., 1996) and interacting directly or indirectly with membranes, cytoskeletal proteins, as well as with small GTPases from the Rab, Ral, and Rho subfamilies and many other proteins in the cell cortex (Wu et al., 2008). As such, the exocyst seems to act as an integrating hub in the cell cortex, mainly in the context of exocytosis. In general, proper exocyst function is essential for polar growth and cell morphogenesis, including invadopodia, lamellipodia, and neuronal dendrites formation in animal cells, bud growth in budding yeast, and cytokinesis in fission yeast (reviewed in Heider and Munson, 2012; Vaškovičová et al., 2013; **Figure 1**). A growing number of papers document functions of the plant exocyst in similar processes with high demand for exocytosis, including root hair growth, hypocotyl cell elongation, cytokinesis, seed coat formation and papilla formation after a pathogen attack in plants (Synek et al., 2006; Hála et al., 2008; Fendrych et al., 2010; Kulich et al., 2010; Pecenkova et al., 2011; Vaškovičová et al., 2013).

## THE EXOCYST AND ACTIN CYTOSKELETON

Deep insight into exocyst functions and their mechanisms came from genetic studies on budding yeast, where the exocyst was originally discovered as a protein complex (Novick et al., 1980; TerBush et al., 1996). In budding yeast cells, secretory vesicles are transported along formin- and Arp2/3-generated actin cables. A common model of the exocyst action suggests that most exocyst subunits arrive to the PM in association with secretory vesicles and cannot localize properly after disruption of the actin cytoskeleton (Boyd et al., 2004; Bendezú et al., 2012). However, Sec3p and part of the Exo70p population can reach its destination, a newly forming bud, independently of the actin cytoskeleton probably via direct association with Rho GTPases (Boyd et al., 2004). Therefore, Sec3p and Exo70p are supposed to act as landmarks of sites for the exocyst localization and action (Finger et al., 1998; Boyd et al., 2004).

Mutations in several exocytosis-related genes cause actin cytoskeleton defects in budding yeast, leading subsequently to impaired cell growth and morphogenesis and also to an mRNA transport and polarization defect that is actin-dependent (Aronov and Gerst, 2004). The identified genes included those encoding *SEC10* and *SEC15* exocyst components and *CDC42* and *RHO3* GTPases regulating the exocyst polar targeting (Wu et al., 2008).

An interesting reciprocal relationship was observed during cell wounding response, where Sec3p and the Bni1p formin are degraded in order to eliminate competition for secretory vesicles required to repair the damaged membrane and cell wall, which are arriving along the pre-polarized cytoskeleton directing current polarized growth. The Bnr1p formin and the Exo70p exocyst subunit relocalize to the damage site followed by redistribution of the Myo2p myosin and delivery of new material (Kono et al., 2012).

In budding yeast, cell polarity and polarized exocytosis is coordinated also by the Rho3p GTPase (Adamo et al., 1999), which can regulate both actin polarity and transport of exocytic vesicles from mother cell to the bud, as well as vesicle docking to the PM. While the Rho3p vesicle delivery function is mediated by Myo2p, the docking requires Exo70p (Adamo et al., 1999).

In the fission yeast *Schizosaccharomyces pombe*, the actin cytoskeleton is dispensable for proper exocyst localization and polarized growth (Bendezú and Martin, 2011; Snaith et al., 2011). While actin-independent polar transport in budding yeasts might be constrained by the narrow bud neck, and bud growth requires motor-driven transport along actin cables, the open cylindrical shape of fission yeast cells may allow actin-independent vesicle transport (Bendezú and Martin, 2011). However, the exocyst and actin cytoskeleton share at least two common upstream regulators – Cdc42 (Estravïs et al., 2011) and Pob1 (Nakano et al., 2011).

The polar exocyst localization and formation of actin cables are dependent on and mutually coupled by Pob1 via its interaction with the For3 formin and the Sec8 exocyst subunit, respectively. Simultaneous deletion of For3 and Sec8 results in isotropic growth, indicating a functional redundancy between microfilaments and the exocyst in cell polarization (Bendezú and Martin, 2011). In contrast, although unable to divide properly, *sec8 exo70* and *sec6 sec8* double mutants are still capable of polarized growth (Bendezú and Martin, 2011).

Although all fission yeast exocyst subunits can localize to cell poles largely independently of the actin cytoskeleton, at least Sec3, Sec5, and Exo70 (most probably as a part of the complete exocyst complex) are more efficiently transported to the cell apex by the Myo52 myosin V along microfilaments (Snaith et al., 2011; Bendezú et al., 2012). Either functional Sec3 or Exo70 is essential for viability and proper localization of other exocyst subunits, suggesting that, as in budding yeast, these two components act as exocyst tethers at the PM (Bendezú et al., 2012). A polarization pathway involving the exocyst relocalization and actin repolarization downstream of Cdc42 also participates in fission yeast mating (Bendezú and Martin, 2013).

Unexpectedly, the fission yeast Sec3 not only acts in exocytosis but also marks sites for actin recruitment and controls overall actin organization via direct binding of For3 (Jourdain et al., 2012). Mutants in Sec3 exhibit lack of microfilaments, depolarized actin patches, and disassembly of the cytokinetic actomyosin ring probably due to a failure in polarization of the For3 formin.

The Exo70 exocyst subunit also interacts both *in vitro* and *in vivo* with the yeast and rat Arpc1/Arc40 subunit of the Arp2/3 complex, a key regulator of actin polymerization. Inhibition of the Exo70 function in rat kidney cells blocks formation of actin-based membrane protrusions and affects cell migration (Zuo et al., 2006), pointing to yet unknown capacity of Exo70 to regulate the actin organization and coordinating thus actin cytoskeleton with membrane trafficking during cell migration. Exo70 was recently shown to promote Arp2/3-driven microfilament nucleation and branching (Liu et al., 2012). Because both the exocyst and Arp2/3 complexes are well conserved across eukaryotes, including plants, their interaction is likely to be conserved as well.

In mammalian cells, actin organization, as well as membrane trafficking, cell growth and differentiation, is regulated by RalA and RalB, ubiquitous small GTPases from the Ras superfamily (Feig et al., 1996). Activated (GTP-bound) RalA forms a stable complex with the exocyst via binding to Sec5 (Brymora et al., 2001; Sugihara et al., 2002; Fukai et al., 2003) and Exo84 (Moskalenko et al., 2003; Jin et al., 2005) exocyst subunits in human and rat cells. Specific inhibition of the Sec5 activity blocks filopodia formation in 3T3 cells, a dynamic process that is highly dependent on actin reorganization and that can be normally induced by RalA or cytokines via Cdc42 (Sugihara et al., 2002). This inhibitory effect could not be attributed to disrupted secretion, since inhibition of secretion by brefeldin A did not affect filopodia formation (Sugihara et al., 2002), indicating that the exocyst-RalA complex may regulate actin reorganization independently of vesicle transport. Both RalA-Sec5 and RalA-Exo84 interactions are necessary for proper regulation of the actin cytoskeleton dynamics, as documented by different morphological consequences of uncoupling these interactions in PC-3 cells, such as defects in lamellipodia formation, rounder cells or extended spindles (Hazelett and Yeaman, 2012). RalA also interacts with the actin cytoskeleton via Myo1c, suggesting its function as a cargo receptor for the Myo1c motor (Chen et al., 2007). Taken together, the exocyst complex as an immediate effector of RalA obviously integrates the secretory pathway and actin cytoskeleton near the PM in mammalian cells (**Figure 1**).

Cells of mouse oocytes can use secretory (Rab11-positive) vesicles associated with the exocyst components via the Rab11–Sec15 interaction (Wu et al., 2005) as adaptable, motorized network nodes regulating the dynamics and density of microfilaments in a myosin Vb-dependent manner (Holubcová et al., 2013). Such an actin modulation is essential for asymmetric positioning of the meiotic spindle and thus for the development of a fertilizable egg in mammals.

Although we can find no dynamic membrane protrusions analogous to filopodia in plant cells, fine F-actin meshwork is essential for polar growth of root hairs, pollen tubes, or stigmatic papillae and this type of growth demanding precise regulation of exocytosis is also strongly dependent on the exocyst function (Yalovsky et al., 2008; Vaškovičová et al., 2013).

Ral GTPases are specific to animals – in plant cells, as in yeast, only homologs to Rho GTPases (called also Rac in animals) are present and due to some plant specific features they are called Rop (Rho of plant). Rop GTPases were clearly implied in the cortical cytoskeleton regulation mostly possibly via plant specific Rop-interacting adaptors (RICs; Fu et al., 2001; Yalovsky et al., 2008). Very significant for the speculations on plant exocyst-cytoskeleton links is a dominant land-plant specific way of Rop activation mediated by specific PRONE-GEF (plant-specific ROP nucleotide exchanger – GDP/GTP exchange factor) regulated by interacting receptor-like kinases (RLKs) that allow for very efficient cortical activation of Rop GTPases in response to plethora of different stimuli including changes in cell wall mechanics (Mucha et al., 2011). Moreover, the first Rop-exocyst interaction observed in plants is not direct – several GTP-bound Rops interact with the Sec3 exocyst subunit in *Arabidopsis* via a plant specific adaptor protein ICR1 which is implied in the regulation of auxin polar transport (Lavy et al., 2007; Hazak et al., 2010; see further). These features along with plant specific transmembrane anchorage of plant F-actin nucleating formins (Cvrčková, 2013; in this issue) indicate that the cortical wiring between actin cytoskeleton and exocytosis in plants will be quite specific.

## THE EXOCYST AND TUBULIN CYTOSKELETON

Microtubules are not essential for exocytosis in budding yeast and no functional link with the exocyst complex has been documented so far (Hammer and Sellers, 2012). In rat kidney cells, however, Exo70 co-localizes with microtubules and the mitotic spindle, and *in vitro*, the exocyst complex reconstituted from recombinant subunits inhibits tubulin polymerization. However, deletions of any of Sec5, Sec6, Sec15, or Exo70 exocyst subunits diminished the inhibition activity. Surprisingly, Exo70 itself could inhibit tubulin polymerization, albeit the exocyst complex lacking the Exo70 subunit did not lose its activity completely. On the other hand, when Exo70 was overexpressed, the microtubule network became disrupted and filopodia-like PM protrusions were formed (Wang et al., 2004).

The protrusion formation is consistent with an observation in *Xenopus* neurons, where a local disassembly of microtubules by focal application of nocodazole induced an addition of a new membrane material at the affected site (Zakharenko and Popov, 1998).

In undifferentiated PC12 neuronal cells, the exocyst complex is associated with microtubules as well as microtubule organizing centers and can be co-immunoprecipitated with microtubules from the total rat brain lysate (Vega and Hsu, 2001). However, upon activation of neuronal differentiation, the exocyst redistributes from perinuclear localization to the growing neurite characterized by high exocytic activity at the PM. The subcellular exocyst localization was affected by treatment with microtubule-disrupting drugs, but not actin-disrupting drugs. These results support a possibility that the exocyst complex acts as a modulator of microtubules to mediate vesicle targeting in animal cells.

It is expected that also in respect to microtubular cytoskeleton-secretory pathway relationship the plant cells will have specific features due to the obvious dependence of the final steps of exocytosis and membrane recycling in plants on the actin cytoskeleton and very possibly exocytosis permissive feature of even dense cortical microtubuli (see below). However, both cytoskeletal systems in plant cells strongly interact (e.g., via specific actin nucleating formins) so that in the real biological context it will be challenging to separate their functions.

## INTERPLAY BETWEEN THE EXOCYST AND BOTH TYPES OF CYTOSKELETON

In contrast to budding yeast, typical vertebral cells use microtubules for long-range cargo transport and microfilaments for short-range transport in cell cortex during later steps of vesicles traffic (Hammer and Sellers, 2012). Several studies pinpoint the potential importance of the exocyst in transition of cargo from microtubules to microfilaments.

Mammalian cell migration involves cooperative reorganization of the actin and microtubule cytoskeletons under the control of Rho GTPases (de Curtis and Meldolesi, 2012). Proper localization and activity of the exocyst is promoted by microtubule-associated GEF-H1, a GTP exchange factor for the RhoA actin activator, in HeLa cells (Pathak et al., 2012). Microtubule depolymerization results in the activation of GEF-H1, which further activates RhoA (Krendel et al., 2002). Importance of this regulation was documented experimentally on the cleavage furrow formation during cytokinesis (Birkenfeld et al., 2007) and on actin dynamics during cell migration (Nalbant et al., 2009). The depletion of GEF-H1 led to accumulation of Rab11-positive secretory vesicles within the cells and to mislocalization of Exo70 and Sec8 exocyst subunits (Pathak et al., 2012). GEF-H1 also directly binds the Sec5 exocyst subunit in a RalA GTPase-dependent manner; the interaction is stronger with free GEF-H1 than with its microtubule associated form (Pathak et al., 2012). The Sec5-GEF-H1 interaction promotes RhoA activation, which then regulates exocyst localization and possibly its assembly, as well as actin polymerization. Exocyst thus first helps to activate RhoA, which subsequently assists functioning of the exocyst, resulting in a positive feedback (Pathak et al., 2012).

Interestingly, despite the different mechanisms of cytokinesis between plants and animals/fission yeast (contraction versus building of a cell plate), the exocyst is involved in both types of cytokinesis (Fendrych et al., 2010).

IQGAP1 is another important regulator of both actin and microtubular cytoskeleton associated with the exocyst. The active RhoA and Cdc42 trigger association of Sec3 and Sec8 exocyst subunits with IQGAP1. This interaction is essential for MT1-MMP protease localization at invadopodia and thus for proper invadopodia functioning (Sakurai-Yageta et al., 2008). IQGAP1 stimulates actin bundling (White et al., 2012) and directly interacts with microtubule plus end binding protein CLIP-170 in neurons (Swiech et al., 2011).

## EXOCYST INTERACTION WITH CELLULAR MEMBRANES

As mentioned earlier, in budding yeast, Sec3p and part of Exo70p population can reach newly forming bud also independently of microfilaments (Boyd et al., 2004). They bind the PM directly via phospholipid phosphatidylinositol 4, 5-bisphosphate (PIP_2_) and indirectly by association with Rho GTPases (He et al., 2007; Zhang et al., 2008; Wu et al., 2010; **Figure 1**). Sec15p binds to the membrane of secretory vesicles via the Sec4p Rab GTPase (Guo et al., 1999) and Sec6p binds Snc2p, a vesicle-associated SNARE protein (Shen et al., 2013). Sec6p also contributes to anchor the exocyst complex at sites of secretion – possibly via interaction with PM-associated proteins (Songer and Munson, 2009). Besides facilitating exocytosis by interactions with Sec9p, a Qbc exocytic t-SNARE protein (Sivaram et al., 2005), and with Sec1, a protein from the Sec1/Munc18 family regulating SNARE functions (Morgera et al., 2012), the exocyst also interacts with the vesicles transporting myosin Myo2p (also a known Sec4p interactor) via the Sec15p subunit that directly binds the motor and allows for its release after vesicle tethering (Jin et al., 2011; Donovan and Bretscher, 2012).

In fission yeast, Sec6 and Sec8 exocyst subunits localize to cell tips largely independent of the actin cytoskeleton, but in a Cdc42 and PIP_2_-dependent manner. Thus, the fission yeast long-range cytoskeletal transport and PIP_2_-dependent exocyst represent parallel morphogenetic modules downstream of Cdc42, raising the possibility of similar mechanisms in other organisms (Bendezú and Martin, 2013). Bendezú et al. (2012) showed that Sec3 and Exo70 tether the exocyst complex arriving with secretory vesicles by direct binding to PIP_2_ and Rho GTPases at the cell poles. In absence of the Myo52 motor protein, vesicles with the entire exocyst can still reach the cell pole by random movement, but less efficiently. In absence of both Sec3 and Exo70, vesicles and the rest of the exocyst fail in delivery and tethering and form aggregates. Also in plants Sec3 subunit of exocyst interacts with membrane lipids (Bloch et al., in preparation).

Very recently Zhao et al. (2013) discovered that Exo70 alone, through an oligomerization-based manner, can generate membrane curvatures *in vitro* independent of the exocyst function. This represents a mechanism creating protrusions even in the absence of actin, albeit it is not clear to what extent stimulated actin polymerization, membrane delivery, and membrane deformation contribute to cell shape changes *in vivo* including formation of membrane protrusions. Thus, Exo70 as a membrane-bending protein may couple the actin dynamics and PM remodeling in morphogenesis.

The exocyst is also essential for large-particle phagocytosis (Mohammadi and Isberg, 2013), Salmonella invasion into host cells (Nichols and Casanova, 2010) and formation of tunneling nanotubes – recently discovered structures connecting cytoplasm of animal cells (Ohno et al., 2010; Mukerji et al., 2012; Schiller et al., 2013). Each of these events could combine all three mechanisms mentioned above. Membrane-deforming ability of Exo70 could function well beyond the cell cortex-associated events, since the exocyst participates in many cellular processes (reviewed in Heider and Munson, 2012; Liu and Guo, 2012).

## PERSPECTIVES ON THE EXOCYST–CYTOSKELETON INTERFACE IN ENDOMEMBRANE BIOGENESIS IN PLANTS

Regulation of the cytoskeleton structure and dynamics in plant cells is very much affected by the cell wall, implying close proximity between secretory pathway, cell wall biogenesis and cortical cytoskeleton. These cellular systems are regulated by small GTPases, especially from the ARF, RAB, and ROP families, major regulators of the cell polarity and morphogenesis closely related to their fungal or animal counterparts (Vaškovičová et al., 2013). Work in the laboratory of Shaul Yalovsky (Lavy et al., 2007; Hazak et al., 2010) showed that the SEC3 exocyst subunit interacts with an activated (GTP-bound) ROP at the PM via ICR1, a founding member of the ICR/RIP protein family (Li et al., 2008; Mucha et al., 2010). RIP3 (also known as MIDD1) interacts in a GTP-bound manner with ROPs and also with the Kinesin-13A to regulate the microtubular dynamics (Mucha et al., 2010). RIP3 is a crucial negative regulator of cortical microtubules in the patterning of secondary cell wall thickening directed by the ROP11 GTPase module (Oda and Fukuda, 2012). At PM sites, where cortical microtubules are locally destabilized, the localized exocytosis-dependent secondary cell wall thickening is blocked (Oda and Fukuda, 2012).

While local destabilization of cortical microtubules seems to stimulate exocytosis in animal cells (see above Zakharenko and Popov, 1998), dense microtubule cortical domains of somatic plant cells are often the cortical domains of highest secretory activity, as in xylem thickening or seed coat epidermal cells with a volcano-like cell wall thickening, where highly polarized delivery of pectins is targeted to extremely dense cortical microtubule domains (McFarlane et al., 2008; Oda and Fukuda, 2012). The exocytosis of pectins into pectin-accumulating pockets depends on exocyst function, implying a possibility that microtubule-rich domains might be a general cortical target recognized by EXO70s or other exocyst subunits, functioning as putative PM landmarks for exocytosis targeting (Žárský et al., 2009; Kulich et al., 2010). Extensive proliferation of the EXO70 gene in land plants (e.g., *Arabidopsis* in endowed with 23 EXO70 paralogs) possibly provides a potential for fine targeting into specific cortical areas (Synek et al., 2006; Cvrčková et al., 2012).

On the contrary, dense cortical microfilament meshwork might block exocytosis in both animal and plant cells (Valentijn et al., 1999; Žárský et al., 2009). For instance, a dense subapical F-actin fringe separating actively growing tip from the rest of the tobacco pollen tube might also be a mechanical obstacle for exocytosis (Lovy-Wheeler et al., 2005). The exocyst is also accumulated at the tip of growing pollen tubes and is obviously involved in exocytosis (Hála et al., 2008). The transport and delivery of secretory vesicles in plant cells is likely to depend on both microfilaments and an interaction of some exocyst subunits with the PM phosphoinositides, like in the case of yeast and animal cells (see above). Phosphoinositide binding was indeed predicted for several *Arabidopsis* EXO70 paralogs based on yeast and animal models (Žárský et al., 2009) and currently proved both biochemically and cytologically in our laboratory for the *Arabidopsis* SEC3 exocyst subunit (Bloch et al., in preparation).

The dynamics of several exocyst subunits at the PM, as monitored by TIRF microscopy in *Arabidopsis* epidermal cells, was unaffected by actin or microtubule cytoskeleton disruption after short (10 min) treatment with inhibitors, however, prolonged actin cytoskeleton disruption (1 h) resulted in exocyst redistribution and aggregation at the PM and impaired dynamics (Fendrych et al., 2013). This is consistent with microfilament involvement not only in the delivery but also in spatial distribution of secretory vesicles and endomembrane compartments (Staehelin and Moore, 1995).

Interestingly, exocyst complexes show almost no lateral movement within the PM in both plant and animal cells, as analyzed by the TIRF microscopy, and very similar time of persistence at the PM of about 10 s was recorded (Fendrych et al., 2013; Rivera-Molina and Toomre, 2013). Similarly, the KAT1 channel is localized inside the PM within positionally stable microdomains, which last, however, for 10s of minutes, in contrast to dynamics of the exocyst (Sutter et al., 2006). It is possible that some transmembrane proteins, e.g., plant-specific transmembrane formins (Martiniere et al., 2011; Cvrčková, 2013; in this issue) create, together with specific membrane lipids, functional clusters stabilized against the lateral movement in the PM. These transmembrane proteins might be immobilized by the binding extracellular domains in the cell wall matrix and provide landmarks for the delivery of secretory vesicles (Martinière et al., 2012).

## CONCLUSION

Direct as well as a circumstantial evidence accumulated over the years concerning interactions and cooperation between the exocyst and cytoskeleton indicates that the exocyst, cytoskeleton, and membrane traffic meet at the active cellular cortex. The exocyst serves an important role in co-ordination of the vesicle trafficking with the cytoskeleton in eukaryotes, in addition to its canonical role in exocytosis. In plant cells, however, we have currently only limited and indirect evidence for this regulatory interplay, urging further research in this direction.

## Conflict of Interest Statement

The authors declare that the research was conducted in the absence of any commercial or financial relationships that could be construed as a potential conflict of interest.
